# Ultra-low-dose CT for extremities in an acute setting: initial experience with 203 subjects

**DOI:** 10.1007/s00256-019-03309-7

**Published:** 2019-09-09

**Authors:** Zlatan Alagic, Robert Bujila, Anders Enocson, Subhash Srivastava, Seppo K. Koskinen

**Affiliations:** 1 Functional Unit for Musculoskeletal Radiology Function Imaging and Physiology, Karolinska University Hospital, Karolinska Vägen Solna, 17176 Stockholm, Sweden; 2 Clinical Science, Intervention and Technology (CLINTEC), Karolinska Institutet, Stockholm, Sweden; 3 Functional Unit for Medical Radiation Physics and Nuclear Medicine, Function Imaging and Physiology, Karolinska University Hospital, Stockholm, Sweden; 4 Department of Physics, Royal Institute of Technology, Stockholm, Sweden; 5 Department of Molecular Medicine and Surgery, Karolinska University Hospital, Karolinska Institutet, Stockholm, Sweden

**Keywords:** Multidetector CT, Trauma, Low-dose CT, Radiography, Wrist injuries/treatment, Ankle injuries/treatment, Emergency radiology

## Abstract

**Objective:**

The purpose of this study was to assess if ultra-low-dose CT is a useful clinical alternative to digital radiographs in the evaluation of acute wrist and ankle fractures.

**Materials and methods:**

An ultra-low-dose protocol was designed on a 256-slice multi-detector CT. Patients from the emergency department were evaluated prospectively. After initial digital radiographs, an ultra-low-dose CT was performed. Two readers independently analyzed the images. Also, the radiation dose, examination time, and time to preliminary report was compared between digital radiographs and CT.

**Results:**

In 207 extremities, digital radiography and ultra-low-dose CT detected 73 and 109 fractures, respectively (*p* < 0.001). The odds ratio for fracture detection with ultra-low-dose CT vs. digital radiography was 2.0 (95% CI, 1.4–3.0). CT detected additional fracture-related findings in 33 cases (15.9%) and confirmed or ruled out suspected fractures in 19 cases (9.2%). The mean effective dose was comparable between ultra-low-dose CT and digital radiography (0.59 ± 0.33 μSv, 95% CI 0.47–0.59 vs. 0.53 ± 0.43 μSv, 95% CI 0.54–0.64). The mean combined examination time plus time to preliminary report was shorter for ultra-low-dose CT compared to digital radiography (7.6 ± 2.5 min, 95% CI 7.1–8.1 vs. 9.8 ± 4.7 min, 95% CI 8.8–10.7) (*p* = 0.002). The recommended treatment changed in 34 (16.4%) extremities.

**Conclusions:**

Ultra-low-dose CT is a useful alternative to digital radiography for imaging the peripheral skeleton in the acute setting as it detects significantly more fractures and provides additional clinically important information, at a comparable radiation dose. It also provides faster combined examination and reporting times.

## Introduction

Missed fractures comprise up to 80% of the missed diagnoses in an emergency department [1]. Early and adequate fracture detection enables effective treatment of patients with shorter hospital stays and a likely decrease in medical costs. Early detection also prevents complications, such as non- or malunion, early osteoarthritis, osteonecrosis, and persistent pain [2]. Plain radiographs are the cornerstone in fracture diagnostics, but computed tomography (CT) is superior for assessing structures of the axial skeleton and extremities, as well as detecting subtle fractures. Arbitrary reformats of CT datasets can be used for determining a more optimal surgical planning and care [3–5]. CT has also been shown to be superior to plain radiographs for musculoskeletal diagnostics in the assessment of fracture healing [6]. Conventional CT is known to give a higher radiation dose (RD) compared to radiographs [7, 8] and to minimize it, ultra-low-dose computed tomography (ULD-CT) has been introduced. The term “ultra-low-dose” refers to an aggressive reduction in RD that is possible by implementing modern iterative reconstruction techniques while still achieving diagnostic image quality [9]. However, reports regarding ULD-CT as a clinical alternative to radiographs in the evaluation of fractures in the peripheral skeleton are lacking. Hence, the aim of this study was to investigate how an ULD-CT protocol for extremities performs in an acute clinical scenario compared to radiographs with respect to diagnostic accuracy, RD, and workflow parameters.

## Materials and methods

The study group included consecutive patients who presented to the emergency department (ED) at the Karolinska University Hospital between December 12, 2017 and October 17, 2018. Inclusion criteria were as follows: age ≥ 16 years, signed written consent, admission from the ED for digital radiography (DR) of a suspected fractured ankle, midfoot or wrist. Exclusion criteria included: < 16 years of age, pregnancy, patients who rejected to provide written informed consent to participate in the study. The final study group comprised 203 patients with a mean (±SD) age of 44.1 ± 16.7 years (range, 18–89 years). A total of 92 (92/203; 45%) patients were males. Four patients underwent a scan of two extremities each, i.e., a total of 207 extremities were included in the study.

Prior to ULD-CT scanning, all subjects underwent DR. Radiographs were acquired using the standard extremity protocol on a Discovery™ XR650 radiography system (GE Healthcare, Buc, France) and a FDR200 AcSelerate™ (Fujifilm, Tokyo, Japan). Ankle-series included four-view examinations (AP, lateral, mortise, and plantar flexion). Foot-series included three-view examinations (AP, lateral, and oblique). Wrist-series included two-view examinations (AP and lateral). Hand-series included three-view examinations (AP, lateral, and oblique). Scaphoid-series included four-view studies (AP, lateral, ulnar deviation external rotation, ulnar deviation internal rotation). Additional views were taken depending on the complexity of the examination, if conventional views failed, or if additional views were requested.

After DR imaging, all subjects were scanned on a Revolution™ CT (GE Healthcare, Waukesha, WI, USA) using an ULD-CT protocol that was developed for this study. This protocol used the iterative reconstruction algorithm ASIR-V (Adaptive Statistical Iterative Reconstruction–V).

For ankle and midfoot examinations, the subjects were in supine position extending the affected leg into the gantry while the contralateral leg was kept outside of the gantry. For wrist examinations, the subjects were in prone position with the affected arm extended above the head into the gantry. If the subject could not achieve this position, then an alternative scanning position was chosen with the subject in standing position wearing a lead apron extending the affected wrist into the gantry. Patients were instructed to position the affected extremity in the scanner’s head holder. The broad detector coverage of 16 cm was sufficient to cover the anatomical region of interest in one axial scan, i.e., in one rotation. Localizers (scan projection radiographs) were excluded from the protocol and the anatomical region of interest was determined by using only the positioning lasers on the scanner. The imaging parameters for the ULD-CT protocol are displayed in Table 1. The diagnostic yield, RD, and workflow parameters for the same patient were compared between the ULD-CT and the DR.

**Table 1:** Exposure parameters of the Revolution™ CT and the radiography systems

	Revolution™ CT	Discovery™ XR650/ FDR200 AcSelerate™
Scan mode	Axial	Projection
Scan field of view	Small body	Variable
Tube potential	120 kV	50–70 kV
Tube current	10 mA	5–20 mA
ASIR-V level	70%	–
Detector coverage	160 mm	–
Number of rotations	1	–
Rotation time	0.28 s	–
Slice thickness	0.625 mm	–
Overlap	0.3125 mm	–
Hi-res mode	On	–
Reconstruction algorithm	HD detail	–
Number of images obtained	512	–

*kV* kilovolts, *mA* milliampere, *ASIR-V* adaptive statistical iterative reconstruction-V

The DR and ULD-CT images were initially analyzed by on-call staff, which comprised board-certified consultant radiologists as well as radiology residents, in order to reflect a standard clinical scenario as much as possible. The readers independently analyzed the DR and ULD-CT images, and the results from each modality were blinded to the readers. The double-reading was performed by a consultant radiologist. To increase the quality of the reports even further, a third-read was performed by a musculoskeletal and trauma radiologist with more than 15 years of clinical experience. The images were viewed at a clinical PACS workstation (Sectra PACS IDS7, v.19.3, Linköping, Sweden) and all image processing features were allowed to be used, including 2D and 3D reformations.

The diagnostic accuracy for acute fracture detection was assessed by comparing preliminary reports between the DR and the ULD-CT for the same patient using the paired McNemar’s test. Also, an odds ratio for acute fracture detection was calculated between ULD-CT and DR. Additional clinically important information was recorded: soft tissue injuries or findings, additional fracture-related findings (articular involvement or additional fractures), non-acute-fracture-related findings that explained the patient’s symptoms, acute fracture with additional important non-acute-fracture-related findings, and cases where ULD-CT confirmed or ruled out suspected fractures on DR.

The reported dose length product (DLP; mGy*cm) from the CT scanner was recorded, and the effective dose (ED; mSv) for each ULD-CT examination was estimated by multiplying the DLP with a body region-specific conversion factor (mSv/DLP) for extremities [10]. The effective dose for DR was estimated with a conversion factor specific for extremities that transfers the dose area product reported by the DR system to effective dose (mSv). The DR conversion factor was calculated with a computational phantom and the radiation dose estimation software PCXMC 2.0 [11]. During the calculation of the DR conversion factors, technique factors and projections relevant to this study were used. For each patient and extremity, the effective dose from each DR projection was estimated and accumulated. For instance, if a patient underwent a scan of the right wrist, hand, and scaphoid, then the total RD for the DR comprised the sum of all projections. Also, apart from the typical projections, additional projections may be required to completely depict the area of interest. Dose contributions from these additional projections were also included. The mean RD was compared between the two modalities.

The mean examination time as well as the mean time to the preliminary report were compared between the two modalities. Examination start time was defined as the time from when the patient entered the imaging room and the end time was defined as the time when the subject left the room. Total examination time was defined as the difference between examination start time and examination end time. In order to assess the time to preliminary report, two radiologists with > 20 years of experience each in emergency radiology independently reported 50 cases each (ULD-CT + DR). The same list of cases was provided for both radiologists. The cases were anonymized and shuffled. A timer was started when the radiologists opened the examination and stopped when they signed the preliminary report. This time was registered as the time to preliminary report. Also, the mean combined time (examination time + time to preliminary report) was calculated and compared between ULD-CT and DR.

For the assessment of the impact of ULD-CT on treatment choices, one independent orthopedic surgeon with > 15 years of experience in orthopedic trauma surgery, retrospectively, reviewed the reports from the cases where ULD-CT provided additional diagnostic information compared to DR and decided whether this information had an impact on the recommended treatment choices. The orthopedic surgeon could choose between three different treatment options: functional treatment (compression bandage), treatment with cast, or surgical treatment.

SPSS software (IBM Corp, Armonk, NY, USA, version 25) was used to perform the statistical analysis. Formal descriptive statistical analyses were used for between-modality comparisons. The pairwise McNemar’s test was used for dichotomous variables such as the per-patient fracture detection rate between the two modalities. The paired *t* test was used for continuous data with a normal distribution and Wilcoxon paired signed rank test was used for continuous data with a non-normal distribution; 95% confidence intervals were calculated and the level of significance was set at *p* < 0.05. Data were given as mean ± SD.

## Results

**Fig. 1:**
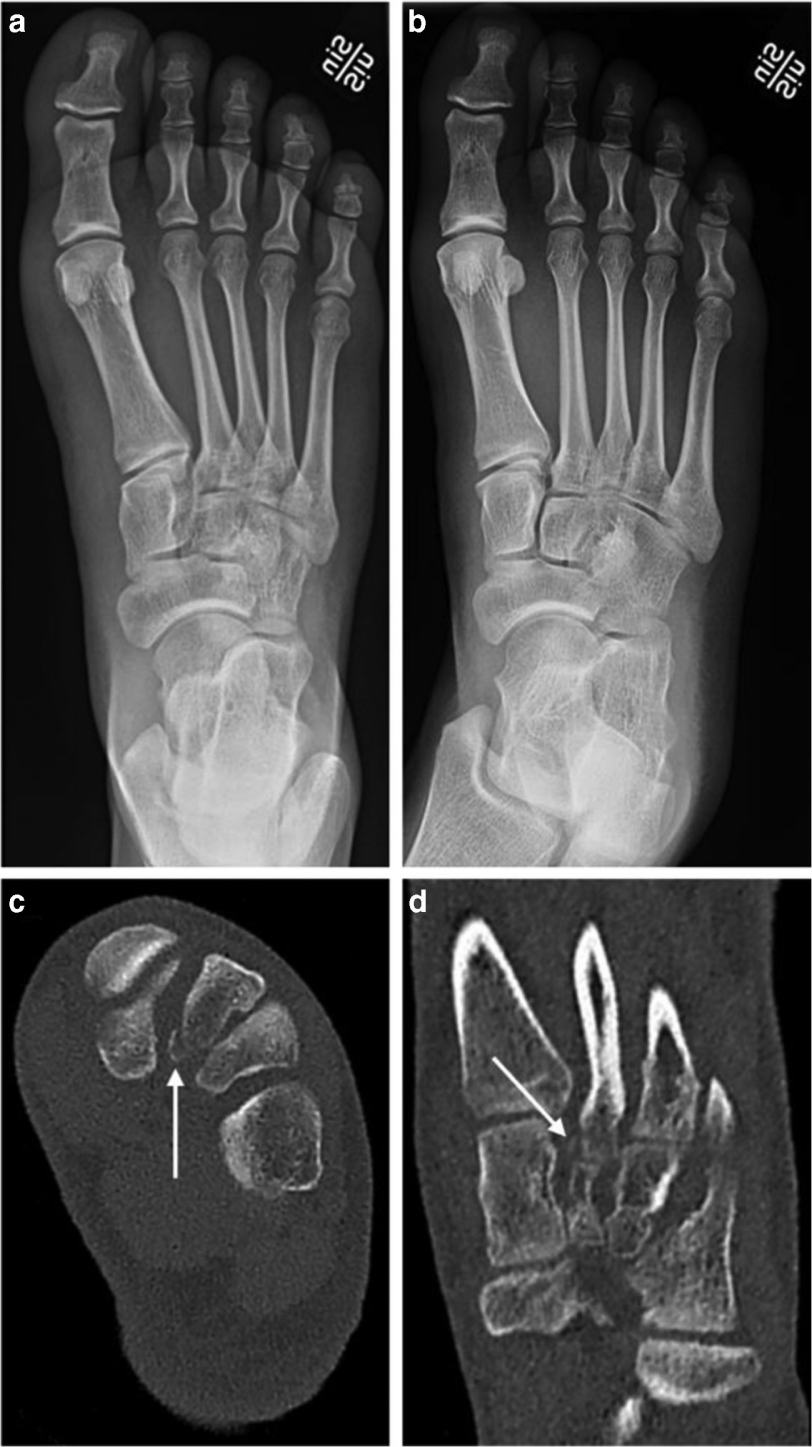
A 28-year-old female fell 2 m from a ladder and landed on her left foot. She presents to the ER unable to bear weight and with pressure pain over tarsals and base of metatarsals. DR AP and AP oblique were negative for fractures (**a**, **b**). ULD-CT demonstrates an avulsion fracture from the plantar aspect of the base of the 2nd metatarsal corresponding to the attachment of the plantar component of the Lisfranc ligament (*arrows*, **c** and **d**), making this a nondisplaced Lisfranc injury. The “fleck sign” is obvious on the oblique coronal ULD-CT image (*arrow*, **d**). The treatment recommendation was upgraded from functional to cast immobilization after ULD-CT

**Fig. 2:**
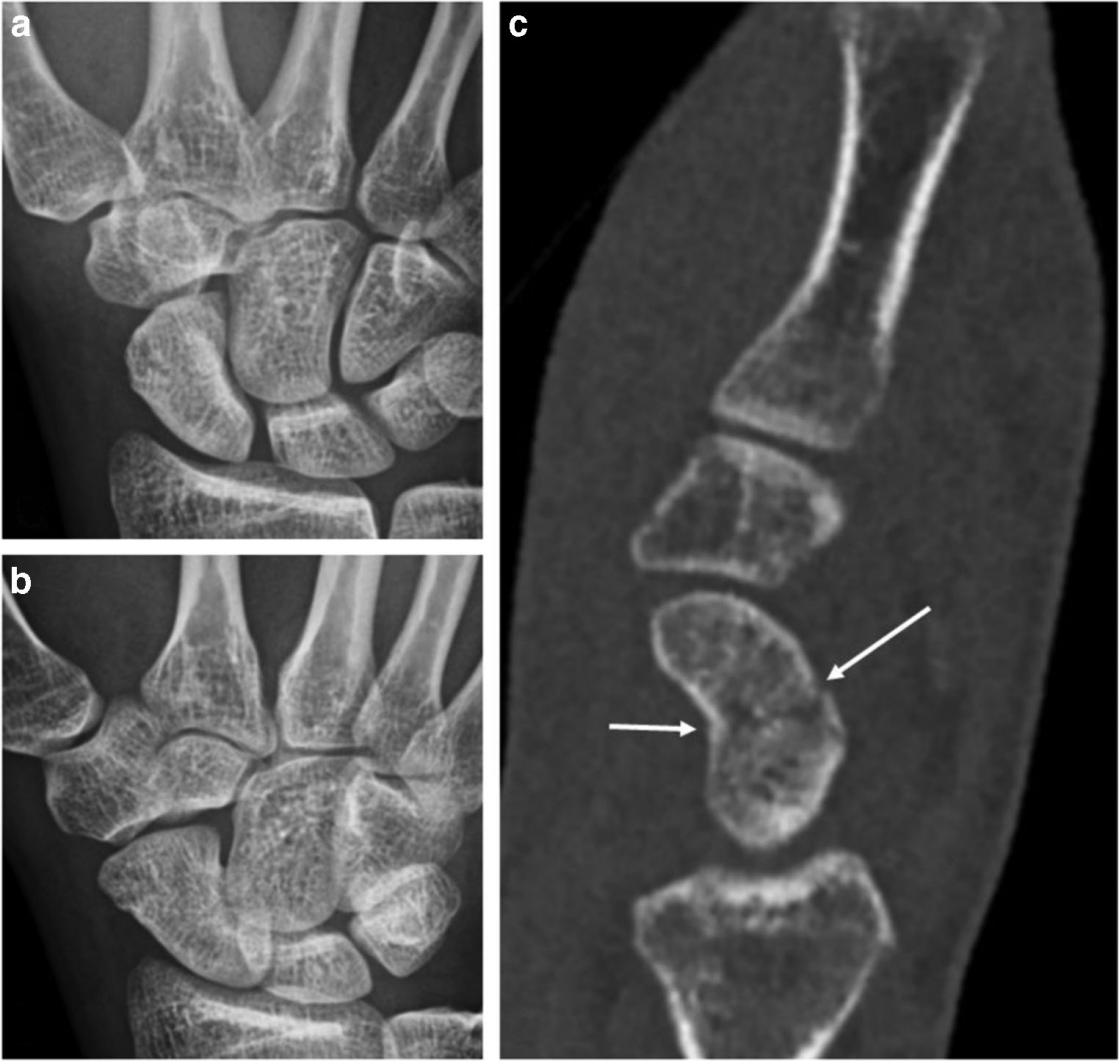
A 29-year-old male fell on an outstretched hand during soccer practice. He experienced instant sharp wrist pain. He presents to the ER with swelling, limited mobility, and local tenderness in the anatomical snuffbox. DR scaphoid projections show no signs of fracture (**a**, **b**). Subsequent ULD-CT demonstrates a nondisplaced scaphoid waist fracture (**c**). The treatment recommendation was upgraded from functional to cast immobilization

Out of 207 extremities in 203 patients, DR and ULD-CT detected one or more acute fractures in 73 and 109 extremities, respectively, and the difference between modalities was statistically significant, *p* < 0.001, (Figs. 1 and 2). The corresponding diagnostic odds ratio for fracture detection for ULD-CT vs. DR was 2.0 (95% CI, 1.4–3.0).

**Fig. 3:**
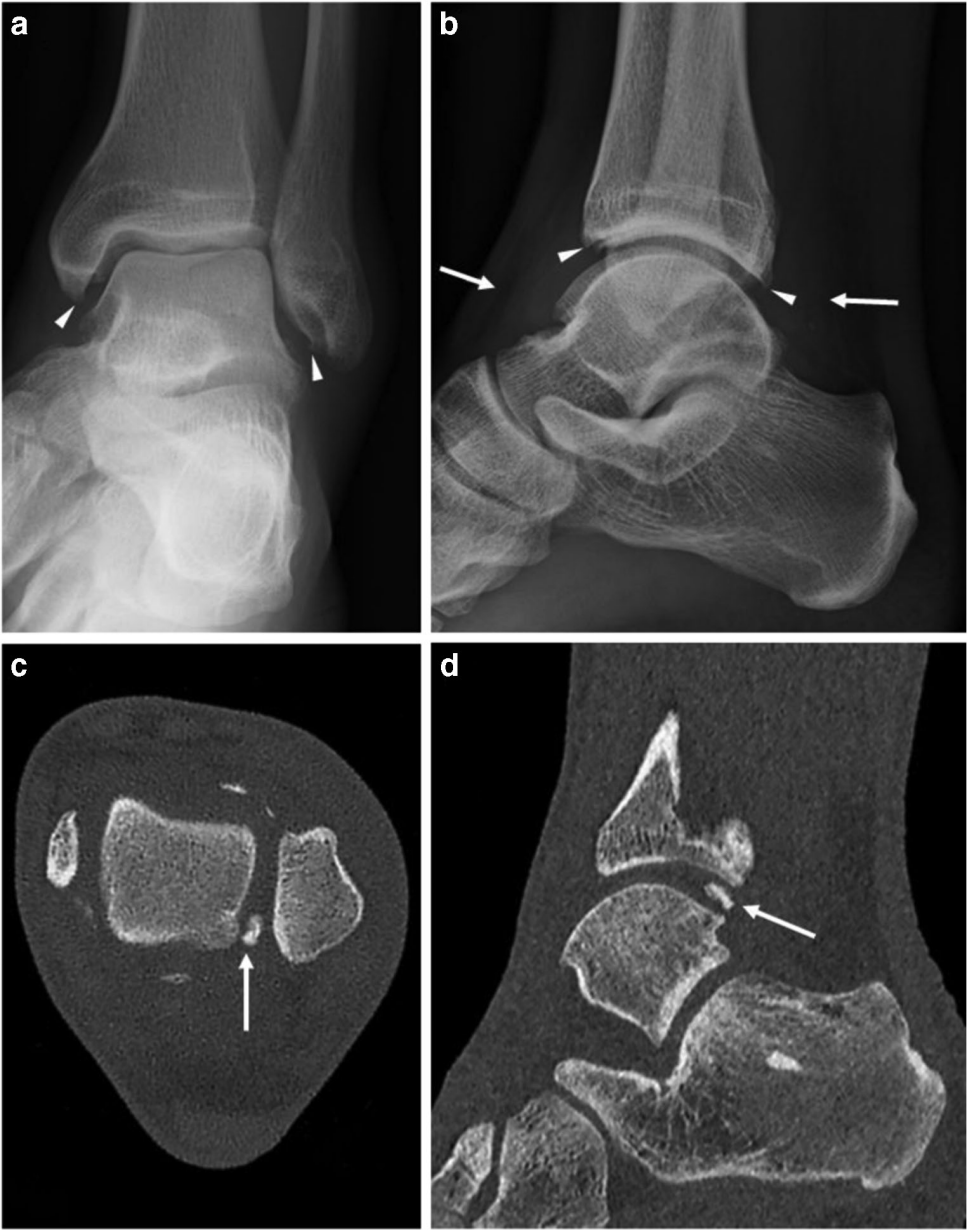
A 31-year-old male that runs several times per week presented to the ER. The same day he experienced a sudden pain in the left ankle and he denies trauma. The left ankle is swollen with limited mobility. The patient is unable to bear weight. Initial DR mortise view and lateral showed signs of early talocrural arthritis with osteophytes surrounding the joint (*arrowheads*, **a** and **b**) as well as a joint effusion (*arrows*, **b**). Subsequent ULD-CT demonstrates a 7-mm loose body in the posterior aspect of the talocrural joint (*arrows*, **c** and **d**). The treatment recommendation was upgraded from functional to surgery for removal of the loose intraarticular body

ULD-CT provided additional fracture-related findings in 33 (33/207; 15.9%) of the extremities where DR could not (Table 2). These findings included additional fractures, articular involvement, and malalignment. In 19 of the extremities (19/207; 9.2%), DR raised the suspicion of a fracture. In ten of these cases, ULD-CT confirmed a fracture and, in the remaining, ULD-CT ruled out a fracture at the suspected site. In two of the extremities (2/207; 1.0%), a fracture was reported on the DR but was subsequently ruled out on the ULD-CT. Furthermore, ULD-CT provided important additional information about non-acute-fracture-related findings that explained the patient’s symptoms in nine cases (9/207; 4.3%), (Fig. 3). Additional diagnostic information provided by ULD-CT is presented in Table 2.

**Table 2:** Additional diagnostic information provided by ULD-CT

Detects an acute fx where DR could not	36 cases (17.4%)
Additional fx-related findings	33 cases (15.9%):
	• 22 cases of additional fractures
	• 9 cases of articular involvement
	• 1 case of increased scapholunar distance
	• 1 case of scaphoid subluxation
Confirmed or ruled out a suspected fx on DR	19 cases (9.2%):
	• 10 ruled out fx
	• 9 confirmed fx
Ruled out a reported fx on DR	2 cases (1.0%)
Differentiated between an acute/old fx where DR could not	4 cases (1.9%):
	• 3 confirmed acute fx
	• 1 confirmed old fx
Soft tissue info	6 cases (2.9%):
	• 2 cases of potential tendon entrapment
	• 1 case of calcifications within the peroneus longus tendon
	• 1 case of chronic rupture of the peroneus longus tendon
	• 1 case of possible pyrophosphate arthropathy
	• 1 case of flexor hallucis longus tenosynovitis
Acute fx with additional significant non-acute-fx-related findings	3 cases (1.4%):
	• 2 cases of older intraarticular loose bodies in the talocrural joint
	• 1 case of chronic osteochondral injury of the talar dome with incipient arthritis of the talocrural joint
Non-acute-fx-related findings that could explain the patient’s symptoms	9 cases (4.3%):
	• 3 cases of radiocarpal arthritis
	• 1 case of a loose body in the talocrural joint with incipient arthritis
	• 1 case of chronic osteochondral injury of the tibia plafond
	• 1 case of subluxation of the MCP-2 joint
	• 1 case of an old avulsion of the base of the scaphoid with increased scapholunate interval
	• 1 case of old fx of the lunate with non-union as well as old fractures of the capitate and hamate with suspected DISI
	• 1 case of chronic osteochondral injury of the talar dome

*fx* fracture, *DR* digital radiography, *MCP* metacarpophalangeal, *DISI* dorsal intercalated segmental instability

The ULD-CT exams had a mean DLP of 2.97 ± 1.67 mGy*cm corresponding to a mean effective dose (ED) of 0.59 ± 0.33 μSv (95% CI, 0.47–0.59), which was comparable to the estimated mean ED of 0.53 ± 0.43 μSv (95% CI, 0.54–0.64) for the DR exams.

The average examination time for ULD-CT was 3.1 ± 1.1 min (95% CI, 2.9–3.2) which was shorter compared to DR 6.0 ± 3.1 min (95% CI, 5.5–6.5) and this difference was statistically significant (*p* < 0.001). Furthermore, the average time to the preliminary report was shorter for DR 1.9 ± 1.2 min (95% CI, 1.6–2.1) compared to ULD-CT 3.6 ± 1.7 min (95% CI, 3.2–3.9) and this finding was also statistically significant (*p* < 0.001). However, the mean combined time (examination time + time to preliminary report) was shorter for ULD-CT 7.6 ± 2.5 min (95% CI, 7.1–8.1) compared to DR 9.8 ± 4.7 min (95% CI, 8.8–10.7) (*p* = 0.002).

Recommended treatment changes after ULD-CT are displayed in Table 3. The total number of extremities with recommended treatment changes after ULD-CT was 34 (34/207; 16.4%) comprising upgraded treatment in 27 (27/207; 13.0%) extremities and downgraded treatment in seven (7/207; 3.4%) extremities. Two extremities were upgraded from functional treatment to surgery and one was upgraded from cast immobilization to surgery. The remainder of the upgraded extremities went from functional treatment to cast immobilization. Out of the 27 upgraded extremities, 18 were due to acute fractures not detected on DR, five were due to additional fracture-related findings, three were due to non-acute fracture related findings that explained the patient’s symptoms, and two were due to suspected fractures on DR that were confirmed on ULD-CT.

**Table 3:** Recommended treatment changes after ULD-CT

Number of extremities	Treatment before ULD-CT	Treatment after ULD-CT
24	Functional*	Cast immobilization
2	Functional*	Surgery
1	Cast immobilization	Surgery
7	Cast immobilization	Functional*

* Functional = compression band

All of the downgraded extremities went from cast immobilization to functional treatment. Out of these, six were due to suspected fractures on DR ruled out on ULD-CT, and one was due to a false-positive fracture reported on DR.

## Discussion

We have demonstrated that ULD-CT is a feasible alternative to DR for imaging the peripheral skeleton in an acute trauma setting. ULD-CT significantly increased the overall fracture detection rate—in 36 extremities (36/207; 17.4%) a fracture was detected with ULD-CT where DR failed to detect a fracture. The diagnostic findings are concordant with a previous study comparing the accuracy of fracture detection between a low-dose CT protocol and conventional radiography for wrist trauma [12]. However, our study has further evaluated the diagnostic performance of a low-dose CT protocol to also include ankle and midfoot trauma, as well as workflow parameters.

Out of the total 207 extremities, there was a recommended treatment change in 34 patients (16.4%) and three of the upgrades were to surgical treatment. This indicates the potential impact of the ULD-CT protocol on the clinical management of patients with suspected fractures in the peripheral skeleton. Also, the ruling out of fractures adds benefit to the patients, since 3.4% of all extremities were downgraded.

Clinical studies have shown that implementation of ASIR-V can decrease the mean RD between 34 and 35% while maintaining image quality [13, 14]. Previously reported mean RD for low-dose CT evaluation of fractures of the peripheral skeleton has been in the range of 10–800 μSv [12, 15–18] and for cone-beam CT (CBCT) in the range of 0.9–14.3 μSv [19]. Our ULD-CT protocol has, to our knowledge, the lowest reported mean RD (0.59 μSv) for the peripheral skeleton in the literature, corresponding to around 1.7 h of exposure to background radiation [20]. This was achieved even though the initial 15 subjects in our study were scanned with a higher RD (up to 2.59 μSv) during the initial development of the ULD-CT protocol. Therefore, the mean RD for the ULD-CT would have been even lower if these 15 subjects would not have been included. However, our experience after this study is that an increase in RD would not have increased the diagnostic accuracy for fracture detection.

The examination times for ULD-CT were significantly shorter compared to DR and the reporting times were longer for ULD-CT compared to DR. However, the combined examination and reporting times were significantly shorter for ULD-CT compared to DR. This implies that the extra amount of electronic information that ULD-CT provides does not lead to a reduction in workflow for acute emergency situations. Our radiographers also experienced the ULD-CT examination to be very convenient, even though no scan projection radiograph was used. They felt confident in using the positioning lasers to set the scan over the area that the patient indicated being the most painful. They also felt that the CT examination was much easier to perform than DR as no specific projections were necessary and also much faster with this single-shot approach. Another experience from the radiographers was that the patients experienced less discomfort and pain during the ULD-CT examination compared to the DR examination since no manipulation of the potentially fractured extremity was required during the ULD-CT scan. Further, we found that casting of an extremity did not obstruct diagnosis using ULD-CT.

The strength of our study was the prospective design in a clinical emergency scenario including a relatively high number of patients. Also, the results of our study have an instant clinical benefit for the patient and the ULD-CT protocol can easily be incorporated into the routine workflow for patients with extremity trauma.

There were several limitations to our study. First, we did not perform any cost analysis for ULD-CT vs. DR. Second, this study was performed at a single site and the patient group was heterogenous. Third, the primary radiology reporting was performed by on-call staff, which comprised board-certified consultant radiologists as well as radiology residents. Fourth, the detector coverage (*z*-axis) was fixed to 16 cm. The first limitation (cost analysis) was not included in the aim of this study, as the primary aim was to investigate whether an ULD-CT protocol is a useful alternative to DR in the evaluation of fractures of the peripheral skeleton or not. A part of the higher costs for CT generally include longer examination and CT review times. As the present study clearly demonstrates, the combined examination and review times for the peripheral skeleton were shorter for CT vs. DR, which could lead to decreased CT costs when scanning the peripheral skeleton. The cost data composes potential groundwork for future studies. The two latter statements reflect the clinical scenario at an emergency radiology department at a university hospital, which could also be regarded as potential strengths of the study in that context. The last statement is one of the prerequisites for the exceptionally low RD. Our experience, after performing this study, is that a detector coverage of 16 cm is sufficient for imaging the peripheral joints. This is the widest detector coverage for axial scanning available on the Revolution™ CT. For wrists, we are confident that an even shorter detector coverage would have been sufficient to cover the joint. However, since no scan projection radiograph was used, we chose 16 cm to safely include the whole joint in the *z*-axis.

The advantage of ULD-CT is that it provides additional clinically important fracture as well as non-fracture-related information, but it is in particular the increased fracture detection rate and the ruling out or confirmation of a fracture that will be of most benefit to the patients. This will lead to less fracture-related complications and decreased society costs in the end.

The disadvantage with ULD-CT is that it is more expensive compared to DR, with roughly twice the cost of a DR exam at our department. ULD-CT also demands more storage volumes, which adds to the costs. Furthermore, many emergency physicians and orthopedic surgeons are viewing the radiology exams themselves before the radiology report arrives and during our study some felt unaccustomed to stacks of CT images compared to planar radiographs. However, they were positively surprised with the increased fracture detection rate of the ULD-CT and most orthopedic surgeons also felt that 3D-rendered images, which the ULD-CT provided, added value in their planning of the surgical approach for open reduction and internal fixation of fractures.

In conclusion, ULD-CT is a useful alternative to digital radiography for imaging the peripheral skeleton in the acute setting, as it detects significantly more fractures and provides additional clinically important information, at a comparable radiation dose. It also provides faster combined examination and reporting times.
